# Application of HPLC–PDA–MS metabolite profiling to investigate the effect of growth temperature and day length on blackcurrant fruit

**DOI:** 10.1007/s11306-018-1462-5

**Published:** 2019-01-08

**Authors:** J. William Allwood, Tomasz L. Woznicki, Yun Xu, Alexandre Foito, Kjersti Aaby, Julie Sungurtas, Sabine Freitag, Royston Goodacre, Derek Stewart, Siv F. Remberg, Ola M. Heide, Anita Sønsteby

**Affiliations:** 1 Environmental and Biochemical Sciences, James Hutton Institute, Invergowrie, Dundee, Scotland DD2 5DA UK; 2 NIBIO, Norwegian Institute of Bioeconomy Research, Pb 115, 1431 Ås, Norway; 3 Department of Plant Sciences, Faculty of Biosciences, Norwegian University of Life Sciences, 1432 Ås, Norway; 4 School of Chemistry, Manchester Institute for Biotechnology, University of Manchester, Princess Street, Manchester, M1 7DN UK; 5 Department of Biochemistry, Institute of Integrative Biology, University of Liverpool, Biosciences Building, Crown Street, Liverpool, L69 7ZB UK; 6 Nofima, Norwegian Institute of Food, Fisheries and Aquaculture Research, 1430 Ås, Norway; 7 School of Engineering and Physical Sciences, Institute of Mechanical, Process and Energy Engineering, Heriot-Watt University, Edinburgh, Scotland EH14 4AS UK; 8 Faculty of Environmental Sciences and Natural Resource Management, Norwegian University of Life Sciences, 1432 Ås, Norway

**Keywords:** Metabolomics, HPLC–PDA–MS, Flavonoids, Anthocyanins, Flavanols, Blackcurrant, Climate, Temperature, Day length

## Abstract

**Introduction:**

Blackcurrant (*Ribes nigrum* L.) is an excellent example of a “super fruit” with potential health benefits. Both genotype and cultivation environment are known to affect the chemical composition of blackcurrant, especially ascorbic acid and various phenolic compounds. Environmental conditions, like temperature, solar radiation and precipitation can also have significant impact on fruit chemical composition. The relevance of the study is further accentuated by the predicted and ongoing changes in global climate.

**Objectives:**

The aim of the present study was to provide new knowledge and a deeper understanding of the effects of post flowering environmental conditions, namely temperature and day length, on fruit quality and chemical composition of blackcurrant using an untargeted high performance liquid chromatography–photo diode array–mass spectrometry (HPLC–PDA–MS) metabolomics approach.

**Methods:**

A phytotron experiment with cultivation of single-stemmed potted plants of blackcurrant cv. Narve Viking was conducted using constant temperatures of 12, 18 or 24 °C and three different photoperiods (short day, short day with night interruption, and natural summer daylight conditions). Plants were also grown under ambient outdoor conditions. Ripe berries were analysed using an untargeted HPLC–PDA–MS metabolomics approach to detect the presence and concentration of molecules as affected by controlled climatic factors.

**Results:**

The untargeted metabolomics dataset contained a total of 7274 deconvolved retention time-*m*/*z* pairs across both electrospray ionisation (ESI) positive and negative polarities, from which 549 metabolites were identified or minimally annotated based upon accurate mass MS. Conventional principal component analysis (PCA) in combination with the Friedman significance test were applied to first identify which metabolites responded to temperature in a linear fashion. Multi-block hierarchical PCA in combination with the Friedman significance test was secondly applied to identify metabolites that were responsive to different day length conditions. Temperature had significant effect on a total of 365 metabolites representing a diverse range of chemical classes. It was observed that ripening of the blackcurrant berries under ambient conditions, compared to controlled conditions, resulted in an increased accumulation of 34 annotated metabolites, mainly anthocyanins and flavonoids. 18 metabolites were found to be regulated differentially under the different daylength conditions. Moreover, based upon the most abundant anthocyanins, a comparison between targeted and untargeted analyses, revealed a close convergence of the two analytical methods. Therefore, the study not just illustrates the value of non-targeted metabolomics approaches with respect to the huge diversity and numbers of significantly changed metabolites detected (and which would be missed by conventional targeted analyses), but also shows the validity of the non-targeted approach with respect to its precision compared to targeted analyses.

**Conclusions:**

Blackcurrant maturation under controlled ambient conditions revealed a number of insightful relationships between environment and chemical composition of the fruit. A prominent reduction of the most abundant anthocyanins under the highest temperature treatments indicated that blackcurrant berries in general may accumulate lower total anthocyanins in years with extreme hot summer conditions. HPLC–PDA–MS metabolomics is an excellent method for broad analysis of chemical composition of berries rich in phenolic compounds. Moreover, the experiment in controlled phytotron conditions provided additional knowledge concerning plant interactions with the environment.

**Electronic supplementary material:**

The online version of this article (10.1007/s11306-018-1462-5) contains supplementary material, which is available to authorized users.

## Introduction

Blackcurrant (*Ribes nigrum* L.) is a perennial shrub native to central and northern Europe and the Russian Federation. It is an important berry crop across the temperate zones of Europe, Asia, New Zealand and to a lesser extent in North America (Hummer and Dale 2010). The chemical composition and quality of blackcurrant berries are known to be influenced by both cultivar properties and environmental conditions (Zheng et al. 2012; Walker et al. 2010; Krüger et al. 2011; Vagiri et al. 2013). During the beginning of fruit growth, the compounds serving as precursors for secondary metabolites are being synthesised. Further, the colour appears, softening begins, ascorbic acid accumulates, and seed fatty acids are produced. At the ripening stage, sugars and phenolic compounds are accumulated, fruits are softer, darker, sweeter and more attractive for seed dispersers. Ripening of the fruit is a complex process, where many physiological and functional changes are precisely controlled by hormonal and signalling pathways under the given environmental conditions (Jarret et al. 2018). According to The Intergovernmental Panel on Climate Change (IPCC) the events of extreme weather or climatic conditions will be more frequent under the future climate scenarios (IPCC 2014). Fruits and berries are important components of the human diet with potential health benefits, it is assumed that climatic disturbances may considerably affect both fruit availability and quality (Moretti et al. 2010).

The effects of environmental conditions on berry quality in field trials may be studied using distinct growth locations (Vagiri et al. 2013) or long-term data series (Zheng et al. 2012), with both experimental approaches potentially leading to similar findings. Accumulation of delphinidin-3-glucoside in blackcurrants grown in Finland showed a positive correlation with summer temperature across the years (Zheng et al. 2012). These results are in agreement with the Swedish study on blackcurrants grown at two latitudinal locations: cool temperatures (northern part of Sweden) vs. a warmer location (southern part of Sweden) (Vagiri et al. 2013), and thereby confirm the role of temperature as a factor influencing the accumulation of individual phenolic compounds in berries. Despite the fact that the environmental impact on chemical composition of blackcurrant has been studied extensively, the specific effects of individual environmental factors on fruit quality are extremely difficult to differentiate because of the interrelation of external stimuli in field experiments (Krüger et al. 2011; Woznicki et al. 2015a). Results of experiments focused upon the environmental effects on various fruit quality attributes are often contrasting, showing distinct responses not only between species, but also among the cultivars and growing sites (Zheng et al. 2018). For example, a negative correlation between ascorbic acid accumulation in blackcurrant and ripening temperature was reported from a field trial conducted in Estonia (Kaldmäe et al. 2013). However, in contrast to these results, temperature (April to July) in the years 1972–2007 was positively correlated with ascorbic acid concentration in blackcurrants grown in Scotland (Walker et al. 2010). In addition, it was observed that significant variations in the accumulation of ascorbic acid took place in blackcurrants grown during the same season in different locations in the UK. Such findings emphasize the high sensitivity of blackcurrants to external conditions.

Therefore, our goal was to perform an experiment in controlled phytotron conditions (Figure S1), which allow the researcher to differentiate the plant responses to individual environmental stimuli, from the uncontrolled environmental factors that classically limit field experiments. The main difficulty in fully controlled experiments with shrubs is to obtain unified plant material. Blackcurrant is a suitable species for such an approach mainly because of the ability to produce single-stemmed plants, which are compact and very similar in size (Sønsteby and Heide 2011). The aim of the present study was thus to investigate in depth the effects of controlled post-flowering temperature and daylength conditions on the accumulation of secondary metabolites using an untargeted approach. Untargeted metabolomics is truly intended for discovery and is not limited to a pre-determined list of metabolites or class of compounds, with the aim to expand the breadth of the metabolome (Allwood and Goodacre 2010; Allwood et al. 2011). Data analysis presented here is focused mainly on molecules significantly affected by the environmental conditions; however, an overview across all annotated compounds may shed additional light on the complexity of the blackcurrant metabolome. In addition, a brief comparison of two analytical approaches (targeted and untargeted) is also presented. A better understanding of the impact of environmental factors on the accumulation of secondary metabolites may facilitate the improvement of production practices and help to enhance future breeding strategies for development of new cultivars better suited to the future climate.

## Materials and methods

### Plant growth and sample collection

Raising and cultivation of blackcurrant plants (cultivar Narve Viking from the Norwegian breeding program) and the physical conditions during the experiment are explained in detail in Woznicki et al. (2015b). In brief, during the last 3 weeks of berry maturation, the plants were exposed to constant temperatures of 12, 18, and 24 °C (± 1 °C) combined with the following photoperiodic conditions: (1) natural long summer day (LD), ca. 18 h (natural LD), (2) 10 h artificial short day (SD), and (3) 10 h SD + 3 h night interruption (SD + NI). Both treatments 1 and 3 were perceived as long day conditions by the plants, but the former also provided a 9% larger daily light integral (total daily photosynthetic active radiation). On the other hand, by using low intensity incandescent lamps for the night interruption (approximately 7 μmol quanta m^−2^ s^−1^), the daily light integral varied by less than 0.5% between treatments 2 and 3, which represent the true photoperiodic test. Plants were also grown outdoors in pots (as a control) under ambient summer conditions (59°40′N). Berries were harvested when fully ripe as judged by berry softness and visual assessment of colour. Berries from one cluster were harvested from the mid-part of two plants into a 50 mL tube and immediately frozen in liquid nitrogen and stored at − 80 °C. The experiment design was fully factorial with a split-plot design, with temperatures as main plots and photoperiod as a subplot. The experiment was replicated with four randomised blocks, each comprising two blackcurrant plants on a separate trolley, giving eight plants per treatment.

### Chemicals

Unless otherwise stated all solvents were of HPLC grade and JT Baker brand (Scientific Chemical Supplies, UK), formic acid was of mass spectrometry grade (Fisher Scientific, UK), morin-hydrate (99% purity) was obtained from Sigma-Aldrich UK, all other reference standards, unless otherwise stated, were obtained from LGC (UK) or extrasynthese (FR). For targeted analysis: Cyanidin-3-glucoside was obtained from polyphenols AS (Sandnes, Norway). Acetonitrile was obtained from VWR International (Fontenay-sous-Bois, France), and water was of Milli-Q quality (Millipore Corp., Bedford, MA, USA).

### Untargeted HPLC–PDA–MS extraction

Freeze dried blackcurrant fruits were homogenised by pestle and mortar. 9 mL of extraction solvent (75% methanol: 24.8% HPLC grade water: 0.2% MS grade formic acid) was added to 300 mg (297–303 mg) of fruit and the sample vortex mixed for 15 s. The samples were next agitated for 30 min with a Heidolph multireax shaker set to speed 10 and centrifuged at 3220×*g* for 10 min with an Eppendorf 5810R at 4000 rpm and 3 °C (rotor A-4-62). 500 µL of supernatant was transferred to each of two 2 mL microcentrifuge tubes (Eppendorf Safe-Lock) per sample extract and dried by speed vacuum concentration at 30 °C for 6 h using the MiVac Duo concentrator system (S.P. Scientific, UK). Preparatory blank extracts were prepared identically, as were 12 extracts of quality assurance (QA) samples that contained an equal mix of all blackcurrant sample materials. Prior to HPLC–PDA–MS analysis, the first set of samples were reconstituted in 250 µL of 20% methanol: 80% water containing 0.5 mM morin to serve as an internal standard, the samples were shaken for 30 min at 2000 rpm on an Ika Vibrax VXR shaker platform, and centrifuged for 10 min at 3 °C and at 18,407×*g* with an Eppendorf 5424R at 14,000 rpm (rotor FA-45-24-11). The extract supernatants were next filtered with 0.45 µm PTFE filter vials (Thomson single step) and transferred to 2 mL HPLC vials with pre-slit caps (Thermo-Fisher, Chromacol 2SVW and 9-SCK(B)-ST1 X, respectively). The samples were stored in the autosampler at 10 °C and analysed within 48 h of reconstitution in positive electrospray ionisation (ESI) mode, after which the ESI source spray cone and ion tube were cleaned, the second set of samples were reconstituted and again analysed within 48 h of reconstitution in ESI negative mode.

### Untargeted HPLC–PDA–MS analysis

HPLC separations were performed with a Thermo Accela 600 HPLC system coupled with an Accela PDA detector (Thermo-Fisher Ltd. UK). The HPLC was operated at a flow rate of 300 µL min^−1^, the column and guard column (Synergi C18 Hydro-RP 80 Ä, 150 × 2.0 mm, 4 µm particle size; Phenomenex Ltd.) were maintained at a temperature of 30 °C. The solvent A, HPLC grade water, and solvent B, HPLC grade acetonitrile, were acidified with 0.1% [v/v] MS grade formic acid. A sample injection volume of 10 µL was employed in full-loop mode. The gradient programme was as follows: hold 2% B 0–2 min, 2–5% B 2–5 min, 5–45% B 5–25 min, 45–100% B 25–26 min, hold 100% B 26–29 min, 100–2% B 26–30 min, hold 2% B 30–35 min. Autosampler syringe and line washes were performed with 8:2 acetonitrile:water. The HPLC column eluent was first monitored by the Accela PDA detector where spectra were collected in wavelength/absorbance mode from 200 to 600 nm with a filter bandwidth and wavelength step of 1 nm, the filter rise time was 1 s, the sample rate was 5 Hz. Additionally three channel set points were employed, Channel A 280 nm, Channel B 365 nm, Channel C 520 nm, with a bandwidth of 9 nm and a sample rate of 10 Hz.

The PDA detector eluent was next transfered to a Thermo LTQ-Orbitrap XL mass spectrometry system operated under Xcalibur software (Thermo-Fisher Ltd. UK). Mass spectra were primarily collected in full scan mode (*m*/*z* 100–2000) at a mass resolution of 30,000 (FWHM defined at *m*/*z* 400) within the FT detector for all samples. Two further methods were applied to obtain ion trees by performing data-dependent analysis (DDA) at MS2 and MS3 levels for the mixed QA samples (Mullard et al. 2015). The first method applied a primary full scan event within the FT, followed by a secondary scan event within the LTQ-IT to collect MS^2^ CID fragmentation spectra for the top three most intense ions as defined within the preliminary full MS scan. The second method was identical, but applied a further tertiary MS scan event where the top three most intense ions detected in each MS2 spectrum, were taken forward for further CID fragmentation and MS3 collection within the LTQ-IT. Helium was applied as a collision gas for CID at a normalised collision energy of 45%, a trapping window width of 2 (± 1) *m*/*z* was applied, an activation time of 30 ms and activation Q of 0.25 were applied, only singly charged ions were selected for DDA, isotopic ions were also excluded. The preliminary full scan event within the FT generated ‘profile’ mode spectral data, whereas the LTQ-IT MS2 and MS3 data were collected in ‘centroid’ mode. To obtain MS2 and MS3 data for as broad range of ions as possible, the DDA MS2 and MS3 methods can be applied several times over restricted mass ranges (e.g. 100–400 *m*/*z*; 400–500 *m*/*z*; 500–600 *m*/*z*; 600–700 *m*/*z*; 700–800 *m*/*z*; 800–1000 *m*/*z*; 1000–2000 *m*/*z*).

A scan speed of 0.1 s and 0.4 s were applied in the LTQ-IT and FT-MS respectively. The Automatic Gain Control was set to 1 × 10^5^ and 1 × 10^6^ for the LTQ-IT and FT-MS respectively. Prior to the analytical run the LTQ-IT and FT-MS were calibrated with the manufacturers recommended calibration mixture and procedures. The following settings were applied to ESI: spray voltage − 3.5 kV (ESI−) and + 4.5 kV (ESI+); sheath gas 60; aux gas 30; capillary voltage − 35 V (ESI−) + 35 V (ESI+); tube lens voltage − 100 V (ESI−) and + 100 V (ESI+); capillary temperature 280 °C; ESI probe temperature 100 °C. For the first 2 min of analysis the eluent flow was directed to waste, whereas from 2 to 29 min the eluent was directed to the MS detector, before being directed back to waste between 29 and 35 min. The samples were analysed in a completely randomised order as two independent analytical blocks respective of ESI positive and ESI negative polarities. For each analytical block, initially eight injections of QA sample were performed for LC–MS system conditioning, after which three further injections of QA sample were performed, followed by six injections of experimental samples and a further QA injection. This was repeated until all samples were analysed, finally the analytical block was concluded with a further two QA injections. A control blank sample was analysed at the start and end of the analytical block, which was finally concluded by collection of the DDA MS2 and MS3 profiles.

### Untargeted HPLC–PDA–MS data processing and peak annotation

The HPLC–PDA–MS raw data profiles were first converted into an MZML centroid format within the Proteowizard (http://proteowizard.sourceforge.net/) MSConvert software package. Each MZML based three-dimensional data matrix (intensity × *m*/*z* × time − one per sample) was converted (or deconvolved) into a vector of *peak responses*, where a *peak response* is defined as the sum of intensities over a window of specified mass and time range (e.g. *m*/*z* = 102.1 ± 0.01 and time = 130 ± 10 s). In this experiment the deconvolution was performed using the freely available XCMS online package (https://xcmsonline.scripps.edu/). XCMS online was operated with the following parameter set points: feature detection; method—CentWave; mass error 5 ppm, minimum and maximum peak width 10 and 60 s respectively, mzdiff 0.01, S/N threshold 6, integration method 1, prefilter peaks 3, prefilter intensity 50,000, noise filter 100,000: RT correction; method—Obiwarp, profstep 1: Alignment; minfrac 0.5, mz width 0.015, bw 5, min samp 1, max samp 100: Annotation; Search for isotopes + adducts, mz absolute error 0.015, ppm error 5.

The XCMS deconvolution results in the production of a Microsoft Excel based XY matrix containing the paired RT and *m*/*z* of each feature, along with the peak intensity in each profiled sample, and where provided adduct and isotope annotations for each *m*/*z*. Applying a set of workflows known as PutMedID (Brown et al. 2009; Allwood et al. 2013), metabolite identifications were made based upon the accurate mass full MS data applying a library of known plant metabolites obtained from the Plant Metabolic Network PlantCyc database (http://www.plantcyc.org) in addition to the Manchester Metabolomics Database (MMD: http://dbkgroup.org/MMD/). Further to performing accurate mass based annotation, the molecular formulae presented for each feature were additionally validated based upon an isotopic peak ratio check performed manually within Xcalibur. The PDA absorbance was also checked against available literature and an inhouse database of soft fruit phenolic compounds, the MS2 and MS3 fragmentation spectra were also matched to the inhouse database of soft fruit phenolic compounds. Where reference standards were available, HPLC RT, high resolution (HR)MS accurate mass, MS2 and MS3 spectra, were all matched to those of the sample extracts, thus providing an MSI level 1 identification (Sumner et al. 2007). Where MS2 and MS3 data were not captured for a given blackcurrant metabolite within this studies sample set, MS2 and MS3 data acquired with previous blackcurrant sample populations were alternatively considered.

### Statistical analysis of untargeted HPLC–PDA–MS datasets

Principal components analysis (PCA) was performed with the SIMCA-P + 12.01 64 bit statistics package. Two PCA-X models were generated based upon the peak ratio (normalised to morin) dataset, the first for the dataset inclusive of QA samples, the second for the dataset after exclusion of blank and QA samples. Prior to PCA, missing values were automatically replaced with a value representative of one-third of the minimum peak ratio across the entire data matrix. PCA scores plots were generated for all possible combinations of PC1-PC5. Complementary PCA loadings plots were also generated for the same PC combinations. The variable identifiers applied within the PCA loadings plots match those given in Table S1 and Table S2. In addition to PCA, univariate statistical analyses were also performed. A non-parametric significance test based upon two-way ANOVA (i.e. the Friedman test) was performed within the MatLab 9.3 2017b software package, a false discovery rate (FDR) correction of 5% was applied based upon the Benjamini–Hochberg procedure. Univariate comparisons were made between blackcurrant fruits grown at 12 °C, 18 °C, 24 °C and ambient temperature, as well as between the variants in daylength (LD, SD, and SD + NI). In addition to conventional PCA, a multiblock hierarchical (H)PCA model was generated according to the methods of Biais et al. (2009), the blocking design investigated the effect of day length (LD, SD, and SD + NI) without regard to cultivation temperature. Metabolite features that were prominent within the conventional PCA loadings as well as showing univariate significance between the temperatures were considered as metabolites significantly changed under the different cultivation temperatures. Metabolite features that were prominent within the multiblock HPCA loadings for the day length block design, as well as showing univariate significance between the different day lengths, were considered as metabolites significantly changed by the day length regime.

### Targeted HPLC–PDA–MS analysis

The targeted HPLC–PDA–MS analysis is previously described within Woznicki et al. (2016). Briefly, blackcurrants (30 g) were homogenized with a blender (Braun MR400, DE), and an aliquot of the homogenate (3 g) was extracted with 1 mM HCl (37%) in methanol (30 mL), followed by sonication for 15 min (Bandelin SONOREX RK 100, Bandelin Electronic GmbH & Co., DE). After centrifugation, the liquid samples were stored at − 20 °C until analysed. The extract of phenolic compounds was filtered through a Millex HA 0.45 µm filter (Millipore Corp., US) before analysis on an Agilent 1100 series HPLC system (Agilent Technologies, DE) equipped with an autosampler cooled to 4 °C, a photo diode array detector, and an MSD XCT ion trap mass spectrometer fitted with an ESI interface. Chromatographic separation was performed on a Synergi 4 μm MAX RP C12 column (250 mm × 2.0 mm i.d.) equipped with a 5 μm C12 guard column (4.0 mm × 2.0 mm i.d.; Phenomenex, US), with mobile phases consisting of A, formic acid/water (2/98, v/v), and B, acetonitrile. The phenolic compounds were identified based on their UV–vis spectra (220 − 600 nm), mass spectra and RT relative to external standards, and comparison with previous reports on phenolic compounds in blackcurrants. The phenolic compounds were classified based on their characteristic UV–vis spectra and quantified by external standards. Anthocyanins were quantified as cyanidin-3-glucoside at 520 nm. All results were expressed as µg per g DW.

## Results and discussion

### HPLC–PDA–MS blackcurrant fruit profiles, metabolite annotation, data complexity and reproducibility

**Fig. 1:**
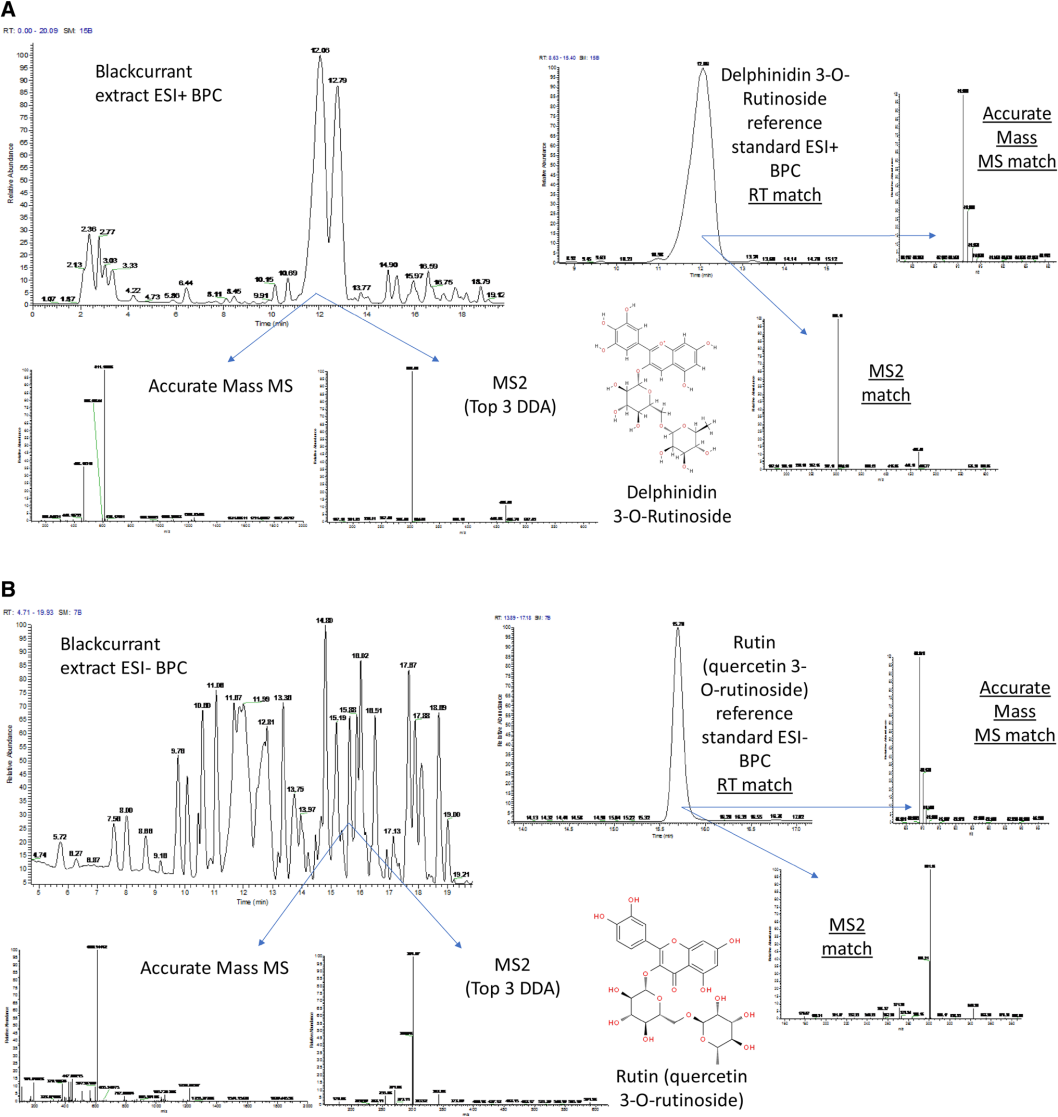
Metabolite annotation in blackcurrant fruit. **a** An example of an MSI level 1 identified compound, delphinidin-3-*O*-rutinoside, in ESI positive mode. **b** An example of an MSI level 1 identified compound, Rutin, in ESI negative mode. *BPC* base peak chromatogram

HPLC–PDA–MS profiling of blackcurrant fruit produces extremely rich metabolite profiles, when the HPLC polar front and the non-polar wash are diverted to waste, as in this study, the profiles are dominated in ESI positive mode by flavonoids such as anthocyanins (Fig. 1a) and in ESI negative mode by flavonoids such as kaempferols and quercetins (Fig. 1b). The deconvolution of these HPLC–PDA–MS profiles within XCMS online, results in the generation of highly information rich datasets. After the removal of features eluting within the first 2 min and final 6 min of the chromatogram, as well as removal of peaks that were dominant within blank sample extracts (more than 2× more intense than the peaks highest intensity within a biological sample) (Di Guida et al. 2016), the ESI positive mode dataset contained a total of 3203 deconvolved RT-*m*/*z* pairs, and the ESI negative mode dataset a total of 4071. The datasets were next subjected to automated peak annotation workflows within PutMedID (Brown et al. 2009). Pearson correlations were first calculated within a ± 10 s moving RT window, peaks that showed a high level of Pearson correlation (greater than 0.8) were grouped as *m*/*z* features that were likely associated with the same compound (i.e. an *m*/*z* group). Accurate mass differences between *m*/*z* within each peak group were next calculated to allow the annotation of the parent *m*/*z*, isotope and adduct ions, as well as common in-source fragments. The neutral accurate mass is next calculated for each RT-*m*/*z* pair and in turn matched to a library of possible molecular formula(s) and associated metabolite name(s). Where the same neutral accurate mass is calculated across multiple adducts in both the positive and negative ion modes for a given metabolite, much higher levels of confidence are instilled that the adduct ions have been accurately annotated and that the correct neutral accurate mass has been attained.

Annotation of metabolites based upon high resolution (HR)MS accurate mass data provides an initial indication of potential identification(s) and a match to potential molecular formula(s), as defined by the metabolomics standards initiative (MSI) as being a level 2 based identification, or in the case of *m*/*z* matched to multiple molecular formulas and identifications but within a single class of metabolites as a level 3 identification, with unknown features classed as level 4 (Sumner et al. 2007). Identification of *m*/*z* features based upon accurate mass alone, does not account for the HPLC RT of the compound, the UV–vis absorbance, or the MS2 and MS3 spectral data. Blackcurrant being a sample matrix that is particularly rich in anthocyanins and flavanols, can be extremely challenging when it comes to metabolite identification, especially when applying accurate mass based annotation alone. This is due to the blackcurrant matrix containing very high numbers of isomeric compounds that are annotated with the same molecular formula and matched to the same metabolite identifications through such an approach. Only by taking other orthogonal data such as the RT, UV–vis absorbance, MS2 and MS3 ion trees, in both positive and negative ion modes, into account, is it possible to characterise such compounds with higher confidence. Limitations in the availability of phenolic reference standards is a further restriction to successful metabolite identification in blackcurrant and other berry fruits.

In this study, once the accurate mass based annotation and molecular formula were proposed for each detected RT-*m*/*z* pair, the molecular formulae were further validated based upon an isotopic peak ratio check performed manually within Xcalibur, the UV–vis absorbance information as well as the MS2 and MS3 fragmentation spectra were also matched to an inhouse database of soft fruit phenolic compounds. Where reference standards were available, HPLC RT (within ± 0.2 min), HRMS accurate mass (to four decimal places), MS2 and MS3 spectra (unit mass and peak ratio match), were all matched to those of the sample extracts (run under identical HPLC conditions), thus providing an MSI level 1 identification (Fig. 1a, b). Where MS2 and MS3 data were not captured for a given blackcurrant metabolite within this study, data acquired with previous blackcurrant sample populations analysed under identical conditions were alternatively considered. Where a given compound ionised in both negative and positive ion modes and different adduct species were observed for each, the MS2 and MS3 spectral data for each ion species were manually assessed and compared. Where data for multiple ion modes and adducts complimented and corroborated each other, much greater confidence was gained that the identification was precise. One example where comparing the MS2 and MS3 ion trees between ionisation modes aided annotation was in the case of the anthocyanin, petunidin-3-*O*-rutinoside, the ion trees and neutral losses were in this case different between ion modes, but still each corroborated the overall compound structure and co-aided in making the final identification. Collection of spectral trees, or at least data to a greater MS level than MS2, is essential to identify complex flavanoid structures, in most cases MS2 provides little information beyond the mass of the compounds sugar moiety, with MS3 being required to fragment the compounds core structure and reveal sub-structural information. However, even ion trees and high levels of MS analysis are not always enough to make an MSI level 1 identification, for example, multiple flavonoids of identical core structure, but with different C6 sugar moieties, or the same sugar moiety but in different bond positions exist in nature. To be able to differentiate such isomers successfully, either extremely high levels of MS analysis (MS4–10) are demanded, or a more likely requirement is the isolation of the target compound through fractionation followed by 2D-NMR analyses, especially in the case of defining bond position. Post annotation, all grouped RT-*m*/*z* pairs, whether a compound identification had been achieved or not, were taken forward, non-grouped low intensity RT-*m*/*z* pairs which could not even be correlated to a single isotopic peak, were not taken forward. Finally, within each dataset, the highly correlated grouped *m*/*z* were further filtered to remove redundant isotope and adduct features, thus further assisting in reducing data complexity and aiding downstream interpretation. The filtered ESI positive and negative mode datasets contained 199 and 350 RT-*m*/*z* pairs, respectively.

The positive and negative ESI datasets were then combined into a single XY matrix, the raw integrated peak areas were normalised to the M+H or M−H signal of the morin internal standard, thus providing a peak response ratio for each feature. Since morin is non-endogenous to blackcurrant but is representative of the flavanoid classes of compounds detected within soft-fruit species, it is routinely applied as an internal standard within our studies. The 549 annotated RT-*m*/*z* pairs were next quality assured, RT-*m*/*z* pairs showing a greater than 20% relative standard deviation (RSD) across the 11 injections of QA sample within the ESI positive and negative mode analytical blocks, were filtered. In addition to QA, the RT-*m*/*z* pair annotations were finally manually checked and any features deemed to be in-source fragments were further filtered out. The QA procedure filtered a total of 48 RT-*m*/*z* pairs, with a further 32 RT-*m*/*z* pairs deemed as being in-source fragments also being filtered. Following these procedures, a total of 469 endogenous blackcurrant compound features were taken forward to statistical analysis (Table S1; Table S2): 53 had been identified to MSI level 1 based upon RT, HRMS accurate mass, MS2 and MS3 matching to authentic reference standards either within this study or previous inhouse blackcurrant studies; 87 had been identified to MSI level 2 by matching of HRMS accurate mass data; 15 were classed as level 2+ where in addition to an HRMS accurate mass match, the MS2 and MS3 data fitted the proposed compounds structure or matched reference MS2 spectra within the MassBank database (https://massbank.eu/MassBank/), but where reference standards were unavailable for confirmation; finally, 221 RT-*m*/*z* pairs were classed as MSI level 3 identifications where a feature could only be broadly identified within a class of metabolites or matched to multiple isomers and 93 were MSI level 4 unknowns.

### Statistical analysis of HPLC–PDA–MS blackcurrant fruit profiles

**Fig. 2:**
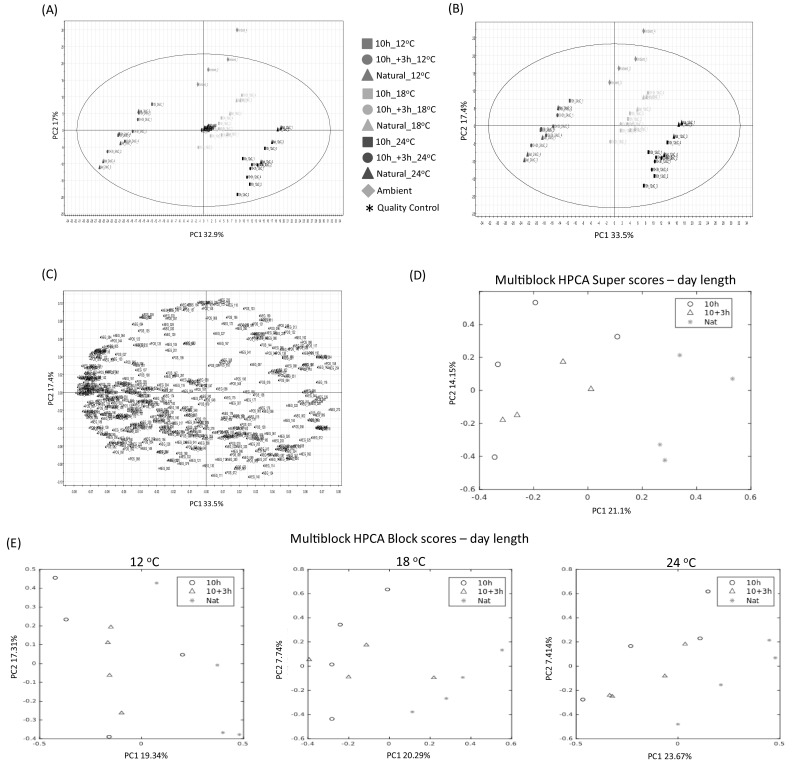
Multivariate statistical analysis of HPLC–PDA–MS non-targeted profiles. **a** Conventional PCA scores plot inclusive of quality assurance samples. **b** Conventional PCA scores plot with quality assurance samples excluded. **c** Conventional PCA loadings plot with quality assurance samples excluded (please refer to Table S1 and S2 for metabolites associated with unique reference numbers). **d** Multiblock hierarchical (H)PCA super-scores plot. **e** Multiblock hierarchical (H)PCA block-scores plots based upon daylength condition. Natural, 10 h, 10 h + 3 h, refer to the following daylength condition descriptions, (1) natural long summer day (LD), ca. 18 h (natural LD), (2) 10 h artificial short day (SD), and (3) 10 h SD + 3 h night interruption (SD + NI), respectively

As a first step to uncovering which of the metabolites (RT-*m*/*z* pairs) were significantly different under the various growth temperature and day length regimes, a conventional PCA model was generated based upon the quality assured dataset containing 469 endogenous blackcurrant metabolites. The first PCA model was inclusive of all samples including the quality assurance profiles, but not the blank profiles to prevent skewing of the model (Fig. 2a). The centralised and co-clustered QA sample profiles within the PC1 × PC2 scores plot at (0,0) (Fig. 2a) is indicative of a very high-quality dataset. A second PCA model was generated where the quality assurance samples were excluded (Fig. 2b). Within the second model, PC1 accounted for 33.5% total explained variance (TEV) and PC2 accounted for 17.4% TEV. The major factor influencing the model is that of growth temperature, with the fruits matured at 12, 18 and 24 °C under controlled conditions being linearly separated from the positive to the negative axis of PC1, the fruits grown in ambient conditions were distinguished from those cultivated under controlled conditions along the PC2 axis (Fig. 2b). The PC1 loadings associated with blackcurrant metabolite changes with increasing cultivation temperature, as well as the PC2 loadings distinguishing ambiently cultivated fruit from control cultivated, were extracted from the PCA loadings plot for the second PCA model (Fig. 2c; refer to Table S1 and Table S2 for metabolite identifications associated with the unique identifiers applied within the PCA loadings plot). The extracted PCA loadings were only considered as statistically significant if they also passed a univariate significance test (Friedman test) following a 5% FDR correction (Table S1 and Table S2). A total of 365 metabolites were deemed to be significant with respect to cultivation temperature based upon the Friedman test, 354 of which were also selected within the PCA loadings and were further investigated. In addition to conventional PCA, a multiblock HPCA model was generated according to the methods of Biais et al. (2009), with a single blocking statement investigating the effect of day length [natural long summer day (LD), 10 h artificial short day (SD), and 10 h SD + 3 h night interruption (SD + NI)], without regard to cultivation temperature. The super scores and individual block scores plots for PC1 × PC2 are presented in Fig. 2d, e, respectively. Based upon the HPCA loadings, a total of 18 metabolites were found to be regulated differentially under the different day lengths, all 18 metabolites also passed the univariate significance test (Friedman test) following 5% FDR correction (Table S1 and Table S2).

### Metabolites up-regulated under high cultivation temperature

**Fig. 3:**
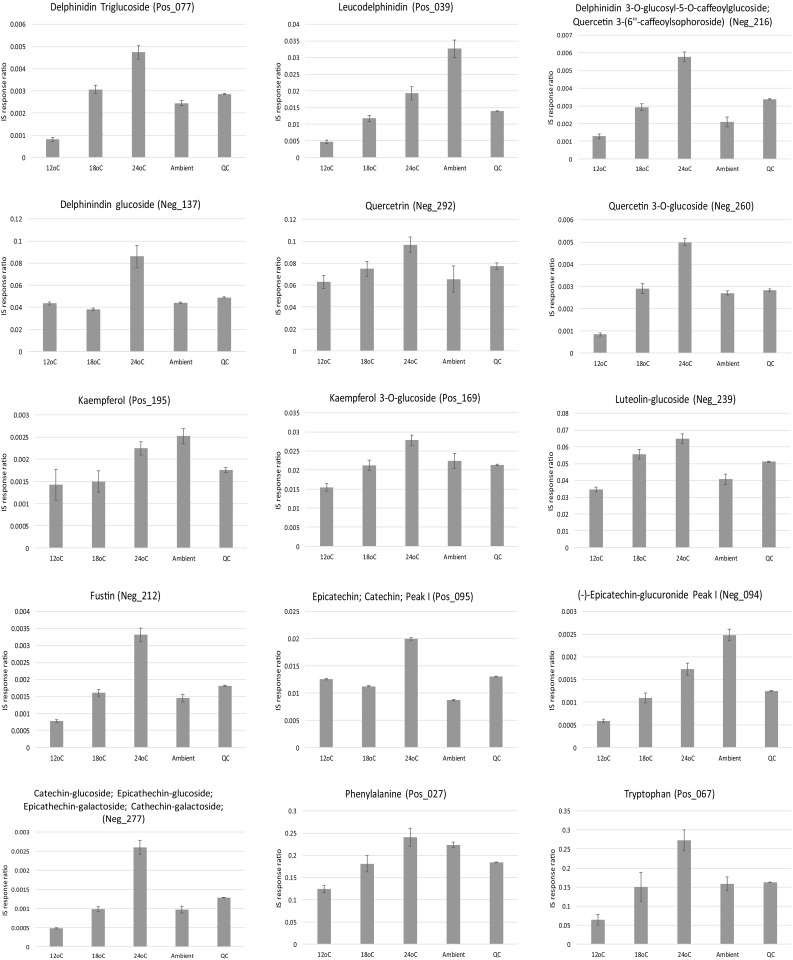
Bar charts of select metabolites that were elevated under increased growth temperatures. Error bars represent the standard error

Based upon the 354 metabolites that were statistically significant within the conventional PCA loadings (Fig. 2b, c; Table S1; Table S2) and which also passed the Friedman significance test, upon further inspection, 100 annotated metabolites showed linear step-wise increases in-line with increasing cultivation temperature. The 100 metabolites that increased in concentration with increasing cultivation temperature represented a diverse range of chemical classes, inclusive of a number of amino acids, organic acids and fatty acids, as well as a large number and diversity of polyphenolic compounds largely representing flavanols, anthocyanins, catechins, terpene derivatives and low MW phenolic acids and derivatives (Fig. 3; Figure S2).

Among all the metabolites upregulated by cultivation temperature, the response of phenylalanine seems to be especially interesting with regard to the secondary metabolism of blackcurrant. This α-amino acid is the primary precursor in the synthesis of flavonoids. Phenylalanine is converted to cinnamic acid by the enzyme phenylalanine ammonia-lyase (Jaakola and Hohtola 2010). Whilst many anthocyanins showed significant differences between cultivation temperatures, only a few anthocyanins showed strong linear responses across the temperature gradient during the phytotron experiment. This indicates that the availability of phenylalanine as the precursor compound is not the limiting factor on the efficiency of the flavonoid biosynthesis pathway. In addition, it is well documented, that anthocyanin accumulation is highly correlated with sugar concentration in fruits (Agasse et al. 2009). Dai et al. (2014) tried to disentangle the physiological relationship between sugars and anthocyanin accumulation, and, surprisingly, observed that the concentration of phenylalanine decreased together with the increasing concentration both of sugars and anthocyanins during in vitro experiments on grapes. This result agrees with our study on blackcurrant, where berries grown in higher temperatures had lower sugar concentration (Woznicki et al. 2017), and as discussed above, had a higher concentration of phenylalanine (Fig. 3).

Tryptophan is another amino acid that was upregulated under higher ripening temperatures (Fig. 3). The two-step conversion of tryptophan to indole-3-acetic acid (IAA, the main naturally occurring auxin) is a key step within the auxin biosynthesis pathway that plays an essential role in many developmental processes. Exogenous application of tryptophan increases the auxin level in plant tissues (Mustafa et al. 2018). In addition, auxins are known to be repressors of ripening as observed in grapes (Ziliotto et al. 2012). Interestingly, Woznicki et al. (2015b) showed an inhibited process of ripening (colouring) of blackcurrants with increasing growth temperature. It can be speculated, that there is a relationship between increased tryptophan accumulation under increased growth temperature, auxin production and ripening of the berries during the experiment.

### Metabolites up-regulated under low cultivation temperature

**Fig. 4:**
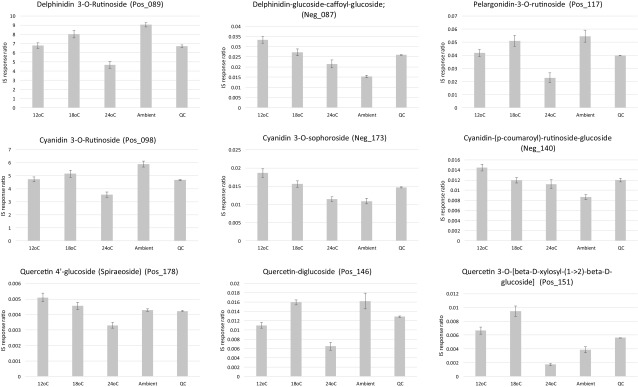
Bar charts of select metabolites that were reduced under increased growth temperatures. Error bars represent the standard error

Taking into consideration the 354 metabolites that were statistically significant within the conventional PCA loadings (Fig. 2b, c; Table S1; Table S2) and which also passed the Friedman significance test, upon further inspection, 42 annotated metabolites showed linear step-wise decreases in concentration in-line with increasing cultivation temperature. The 42 metabolites that decreased in concentration with increasing cultivation temperature (Fig. 4, Figure S3), as for those that increased in concentration with increasing cultivation temperature (Fig. 3, Figure S2), represented a diverse range of chemical classes. Interestingly, the accumulation of the two most abundant anthocyanins in blackcurrant (delphinidin-3-*O*-rutinoside and cyanidin-3-*O*-rutinoside), which are representative of more than 60% of the total anthocyanins, are first slightly elevated between 12 and 18 °C, before being strongly reduced at the highest ripening temperature of 24 °C (Fig. 4).

Similarly, a strong reduction of anthocyanin accumulation under 35 °C heat stress was observed in grapes, as a result of inhibition of mRNA transcription (Mori et al. 2007). It is known that high ripening temperature can be a suppressing factor for the expression of key genes controlling anthocyanin biosynthesis, such as CHS (chalcone synthase), ANS (anthocyanidin synthase), and UFGluT (UDP-glucose:flavonoid 3-*O*-glucosyltransferase) (Ubi et al. 2006). Another reason for lower accumulation of those metabolites under the highest temperature regime might be the degradation of anthocyanins, which was observed previously in the grape cultivars Malbec and Bonarda when cultivated under high temperature conditions (de Rosas et al. 2017).

Many quercetin derivatives showed lower accumulation under increased ripening temperature (Fig. 4). The relationship between temperature during ripening of the berries and flavanols profiles has been less extensively studied. However, Cohen et al. (2008) observed, in agreement with our results, a higher proportion of flavanols with di-hydroxylation, as quercetin, in the cv. Merlot grape under cooler ripening conditions when compared with a higher control temperature.

### The effect of ambient versus controlled growth conditions

**Fig. 5:**
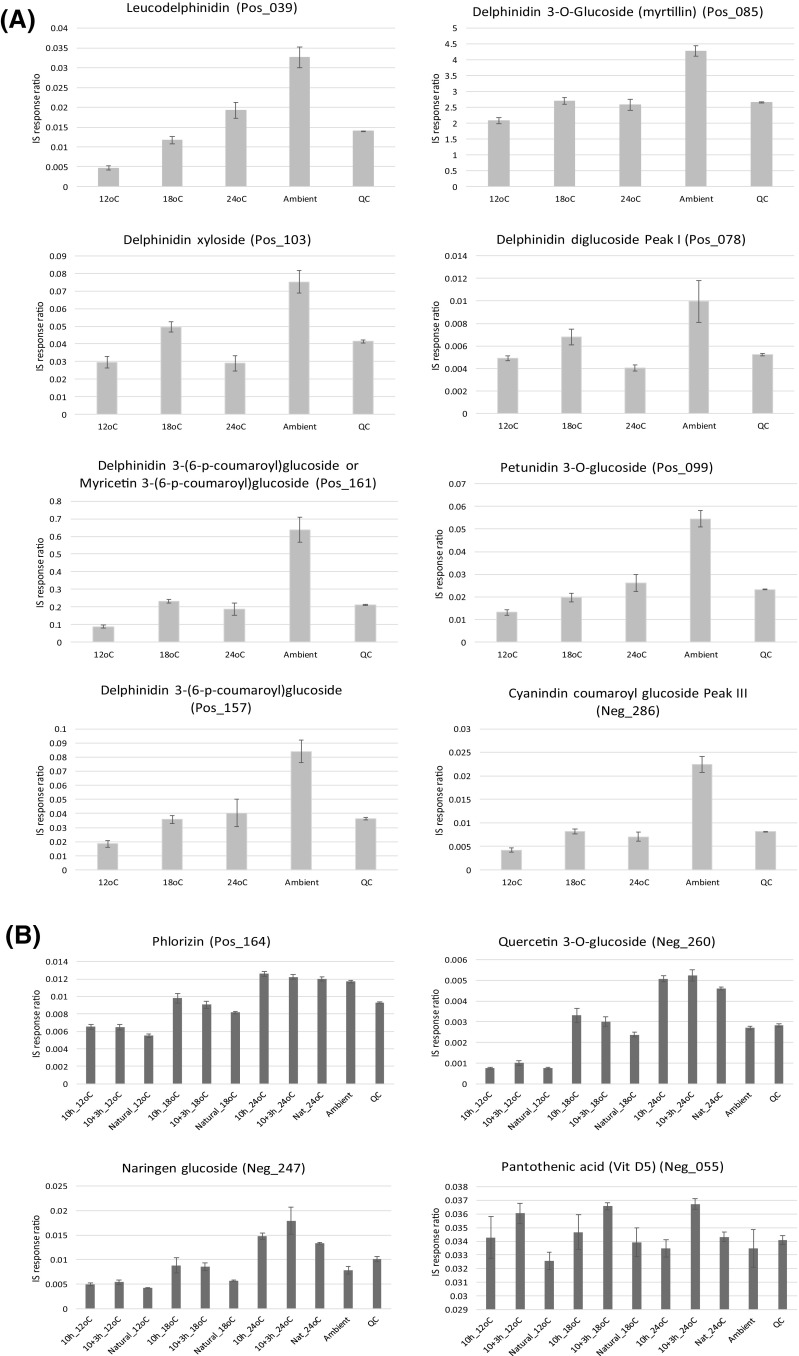
Bar charts of select metabolites that were elevated in ambient growth conditions (**a**), and which were affected by day light conditions (**b**). Error bars represent the standard error. Natural, 10 h, 10 h + 3 h, refer to the following daylength condition descriptions, (1) natural long summer day (LD), ca. 18 h (natural LD), (2) 10 h artificial short day (SD), and (3) 10 h SD + 3 h night interruption (SD + NI), respectively

For further comparisons of growth temperature and daylength, the experimental design also took into consideration the effect of growing blackcurrant fruits in ambient conditions compared to the controlled growth system. In our recent paper (Woznicki et al. 2015b), comparison between average daily mean outdoor (ambient) temperatures during the entire experimental period and constant temperatures in the phytotron compartments during fruit ripening is presented. Interestingly, it was discovered that cultivating the blackcurrant fruits in ambient as opposed to controlled conditions resulted in a further 34 annotated metabolites, which again represented a diverse range of chemical classes, being of far higher concentration under ambient than controlled cultivation conditions (Fig. 5a, Figure S4). The anthocyanins were the most represented chemical class and in some cases could double or more in concentration when the blackcurrant fruits were cultivated ambiently. It is known that accumulation of anthocyanins is mediated by UV-B radiation by affecting the phenylalanine ammonia-lyase enzyme activity, a key enzyme in the flavonoid biosynthesis pathway (Jaakola and Hohtola 2010). In addition, epidermal accumulation of flavonoids, which act as protecting agents against the harmful effects of UV-B radiation, is promoted by the radiation itself (Treutter 2006). Therefore, one of the reasons for higher concentration of the anthocyanins in berries matured under the outdoor conditions compared to the phytotron, might be the significant UV-B radiation blocking properties of the phytotron glass cover. Anthocyanin biosynthesis and accumulation in fruits is also sensitive to day–night temperature fluctuations. It was shown that a single night of chilling temperature enhanced the transcription of MYB10 factor and the biosynthesis of anthocyanins in apples (Lin-Wang et al. 2011). Together with the UV–filtering effect of the phytotron glass cover, this mechanism might have played an important role in generating the higher accumulation of anthocyanins under outdoor conditions.

### The effect of daylength

To investigate the effect of day length (natural LD, 10 h SD, 10 h SD + NI), a multiblock HPCA model was developed (Biais et al. 2009) where the blocking statement separated each cultivation temperature group (12, 18, 24 °C) with the aim of identifying similar patterns between the day length variants. Based upon the HPCA loadings, a total of 18 metabolites were found to be regulated differentially under the different day lengths, all 18 metabolites also passed the univariate significance test (Friedman test) following 5% FDR correction (Fig. 5b, Figure S5). Due to the design of the experiment, where the largest effects are observed for temperature treatments, it is challenging to differentiate the clear effects of daylength treatments. However, some compounds were affected similarly by daylength treatment regardless of temperature conditions. For example, accumulation of naringen glucoside, a flavonoid with potential health benefits commonly found in citrus fruits (Gorinstein et al. 2005; Yáñez et al. 2007), is suppressed in blackcurrant by natural long day Nordic summer conditions at all temperatures (Fig. 5b). In general, it is known that high levels of solar radiation tend to increase flavonoid concentration in fruits, although high variation in response of flavonoids to light has been observed between different fruit species and even between cultivars within species (Zoratti et al. 2014). Interaction of light conditions with other environmental factors can also change the response markedly (Zoratti et al. 2014). An intriguing accumulation pattern was also observed for pantothenic acid. This important vitamin (B5) seems to be relatively stable across the treatments except for in fruit housed within the artificial daylength conditions provided by night interruption using incandescent lamps (Fig. 5b). It can be hypothesised that the high proportion of far-red radiation of this light source might affect the biosynthesis of pantothenic acid, resulting in higher accumulation of this vitamin in the berries.

### A comparison of non-targeted HPLC–PDA–MS profiling and targeted HPLC–PDA–MS

**Fig. 6:**
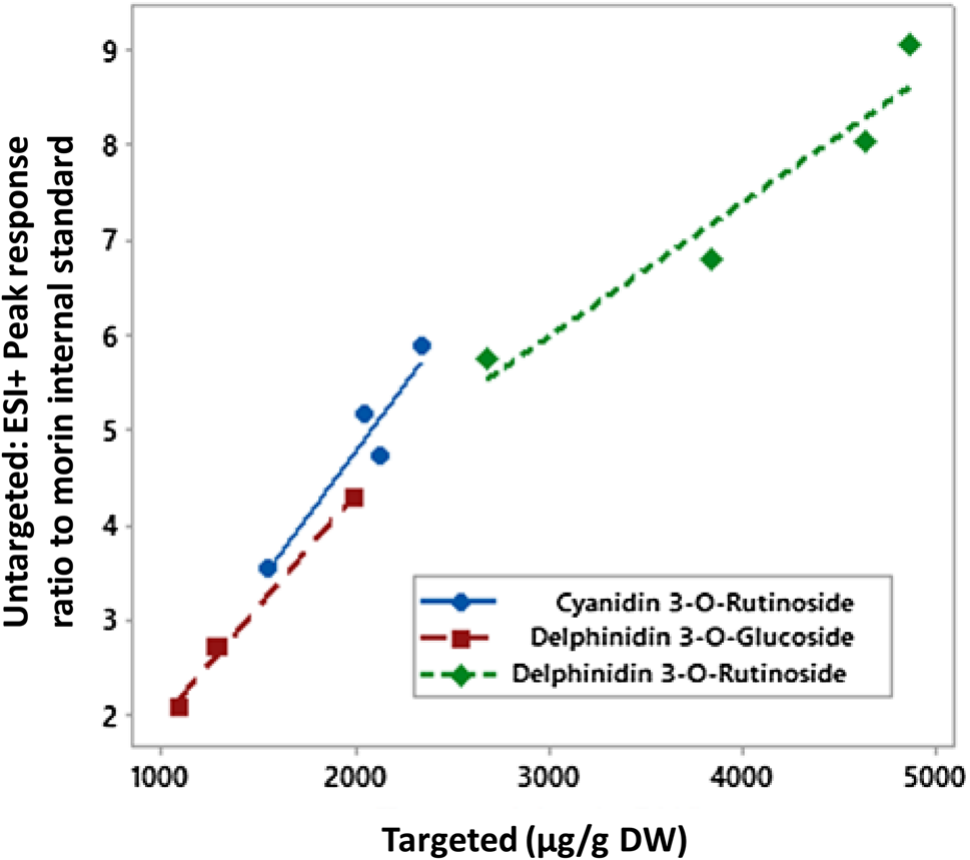
Comparisons of the major blackcurrant anthocyanins as detected by targeted and non-targeted metabolomics analysis

After extraction of detailed results obtained from the cultivar Narve Viking (Woznicki et al. 2016; data presented here is previously unpublished), it is possible to perform a comparative analysis of the two analytical approaches, targeted and untargeted metabolomics analysis. Comparison of the three most abundant anthocyanins in blackcurrant (cyanidin-3-*O*-rutinoside, delphinidin-3-*O*-glucoside and delphinidin-3-*O*-rutinoside) shows an almost linear relation between the two analytical approaches (Fig. 6). Overall correlation between peak response ratios (non-targeted analysis) and exact concentration (targeted analysis) of those compounds in fruits is high (r = 0.968, p < 0.001), indicating that non-targeted metabolomics analysis is suitable for the accurate measurement of relatively low levels of variability in the most abundant anthocyanins of berries rich in phenolic compounds. However, further comparative work examining relations between the two analytical approaches, as well as calibration development, are needed for unambiguous conclusions. Blackcurrant is known to be an excellent source of ascorbic acid (vitamin C) (Hummer and Dale 2010). The concentration of vitamin C varies greatly between cultivars, from 130 mg/100 mL juice to over 350 mg/100 mL juice in some breading lines (Brennan and Graham 2009). Results from the targeted analysis of ascorbate accumulation in blackcurrant cv. Narve Viking from the present study, revealed higher accumulation of both l-ascorbic acid and dehydroascorbic acid at 12 °C compared to 18 and 24 °C (Woznicki et al. 2017). Interestingly, analysis based on the untargeted approach also revealed the same pattern in accumulation of ascorbic acid with relative peak ratios of 0.0062, 0.0048 and 0.0046, for 12, 18 and 24 °C, respectively (Table S1 and Table S2). Despite of the low signal intensity for ascorbic acid in the given method, increased accumulation of this vitamin under low temperature treatment is in agreement with the targeted HPLC–DAD (Woznicki et al. 2017) analysis and confirms the suitability of the untargeted approach.

## Concluding remarks

Blackcurrant maturation under controlled ambient conditions revealed a number of insightful relationships between environment and chemical composition of the fruit. A prominent reduction of the most abundant anthocyanins under the highest temperature treatments indicated that blackcurrant berries in general may accumulate lower total anthocyanins in years with extreme hot summer conditions. A similar trend was observed for vitamin C accumulation. Additionally, repressed accumulation of flavonoids, mainly anthocyanins, under the glass cover in the phytotron should be further investigated, especially in light of the current trend of increased production of fruits and berries in plastic tunnels and greenhouses (Mann 2015). The advantage of applying untargeted metabolomics is the possibility for broad metabolome screening, which can be used for the discovery of novel compounds. For example, the annotated compounds within this study reported a number of chemicals described for the first time in blackcurrant fruit, albeit at a putative level of annotation and which require further comparative analytical work (both multi-stage MS and 2D NMR) with certified reference standards to validate. In conclusion, HPLC–PDA–MS metabolomics is an excellent method for broad analysis of chemical composition of berries rich in phenolic compounds. Moreover, the experiment in controlled phytotron conditions provided additional knowledge concerning plant interactions with the environment. The combination of such controlled experimental conditions and untargeted metabolomics provided results which deepen our understanding of plant phenotypic plasticity and may help to improve production practices and enhance the future breeding strategies for development of better blackcurrant cultivars suited to future climate scenarios.

## Electronic supplementary material

Below is the link to the electronic supplementary material.

Supplementary material 1 (PPTX 1194 KB) Figure S1: Controlled cultivation of blackcurrants within the phytotron

Supplementary material 2 (PPTX 197 KB) Figure S2: Bar charts of all metabolites (not shown in Figure 3) that were elevated under increased growth temperatures. Error bars represent the standard error.

Supplementary material 3 (PPTX 113 KB) Figure S3: Bar charts of all metabolites (not shown in Figure 4) that were reduced under increased growth temperatures. Error bars represent the standard error.

Supplementary material 4 (PPTX 151 KB) Figure S4: Bar charts of all metabolites (not shown in Figure 5a) that were elevated in ambient growth conditions. Error bars represent the standard error.

Supplementary material 5 (PPTX 633 KB) Figure S5: Bar charts of all metabolites (not shown in Figure 5b) that were affected by day light conditions. Natural, 10 h, 10 h + 3 h, refer to the following daylength condition descriptions, (1) natural long summer day (LD), ca. 18 h (natural LD), (2) 10 h artificial short day (SD), and (3) 10 h SD + 3 h night interruption (SD + NI), respectively.

Supplementary material 6 (XLSX 97 KB) Table S1: Friedman significance tests, class averages and associated relative standard deviations for the most statistically significant metabolites. Natural, 10 h, 10 h + 3 h, refer to the following daylength condition descriptions, (1) natural long summer day (LD), ca. 18 h (natural LD), (2) 10 h artificial short day (SD), and (3) 10 h SD + 3 h night interruption (SD + NI), respectively. RSD, relative standard deviation. Unique peak reference numbers applied to PCA loadings plots are also provided.

Supplementary material 7 (XLSX 464 KB) Table S2: Table of all metabolites. Retention Times (RT), accurate mass MS ions, MS2 ions, MS3 ions, morin internal standard normalised peak ratios for all samples, unique peak reference numbers applied to PCA loadings plots. Natural, 10 h, 10 h + 3 h, refer to the following daylength condition descriptions, (1) natural long summer day (LD), ca. 18 h (natural LD), (2) 10 h artificial short day (SD), and (3) 10 h SD + 3 h night interruption (SD + NI), respectively. RSD, relative standard deviation.

## Data Availability

Metabolomics data have been deposited to the EMBL-EBI MetaboLights database (https://doi.org/10.1093/nar/gks1004. PubMed PMID: 23109552) with the identifier MTBLS773. The complete dataset can be accessed here https://www.ebi.ac.uk/metabolights/MTBLS773.
