# Supplementary figures and images for: Application of HPLC–PDA–MS metabolite profiling to investigate the effect of growth temperature and day length on blackcurrant fruit
Source: Metabolomics. 2019 Jan 8;15(1):12. doi: 10.1007/s11306-018-1462-5 (PMC6326004; doi:10.1007/s11306-018-1462-5)

## Slide 1
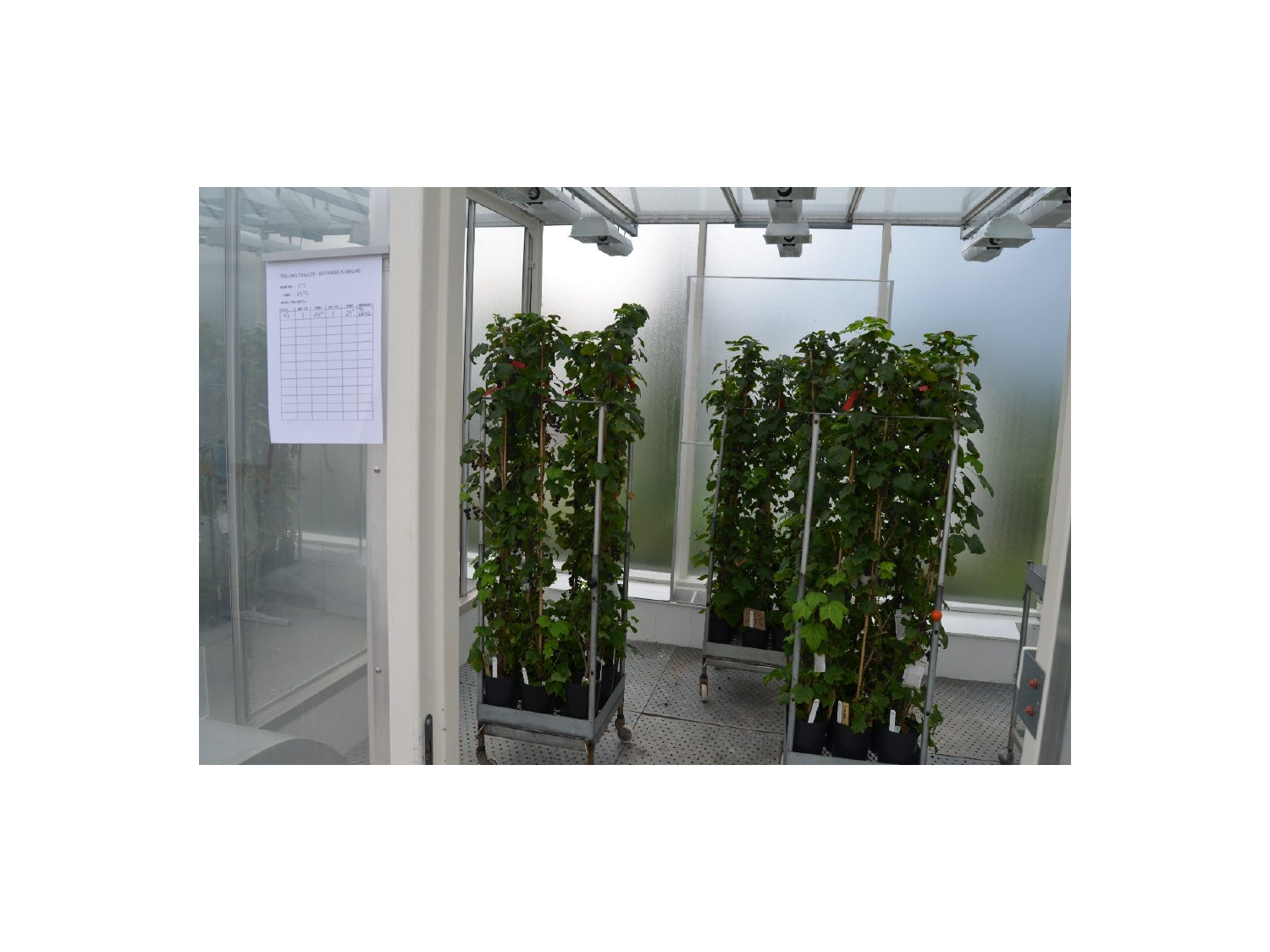

Supplement: Supplementary file 1 — Supplementary material 1 (PPTX 1194 KB) Figure S1: Controlled cultivation of blackcurrants within the phytotron [file 11306_2018_1462_MOESM1_ESM.pptx]

## Slide 1
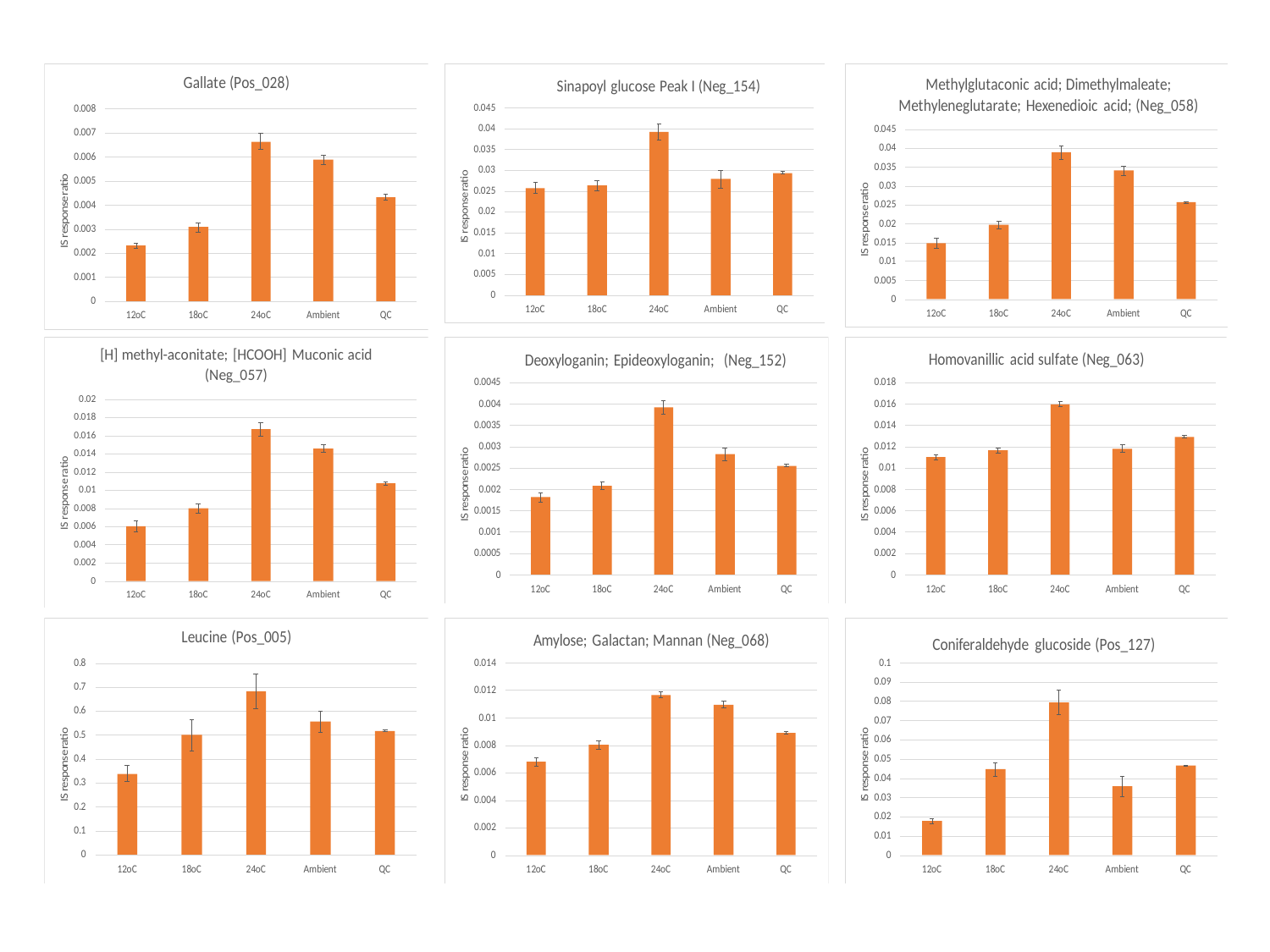

## Slide 2
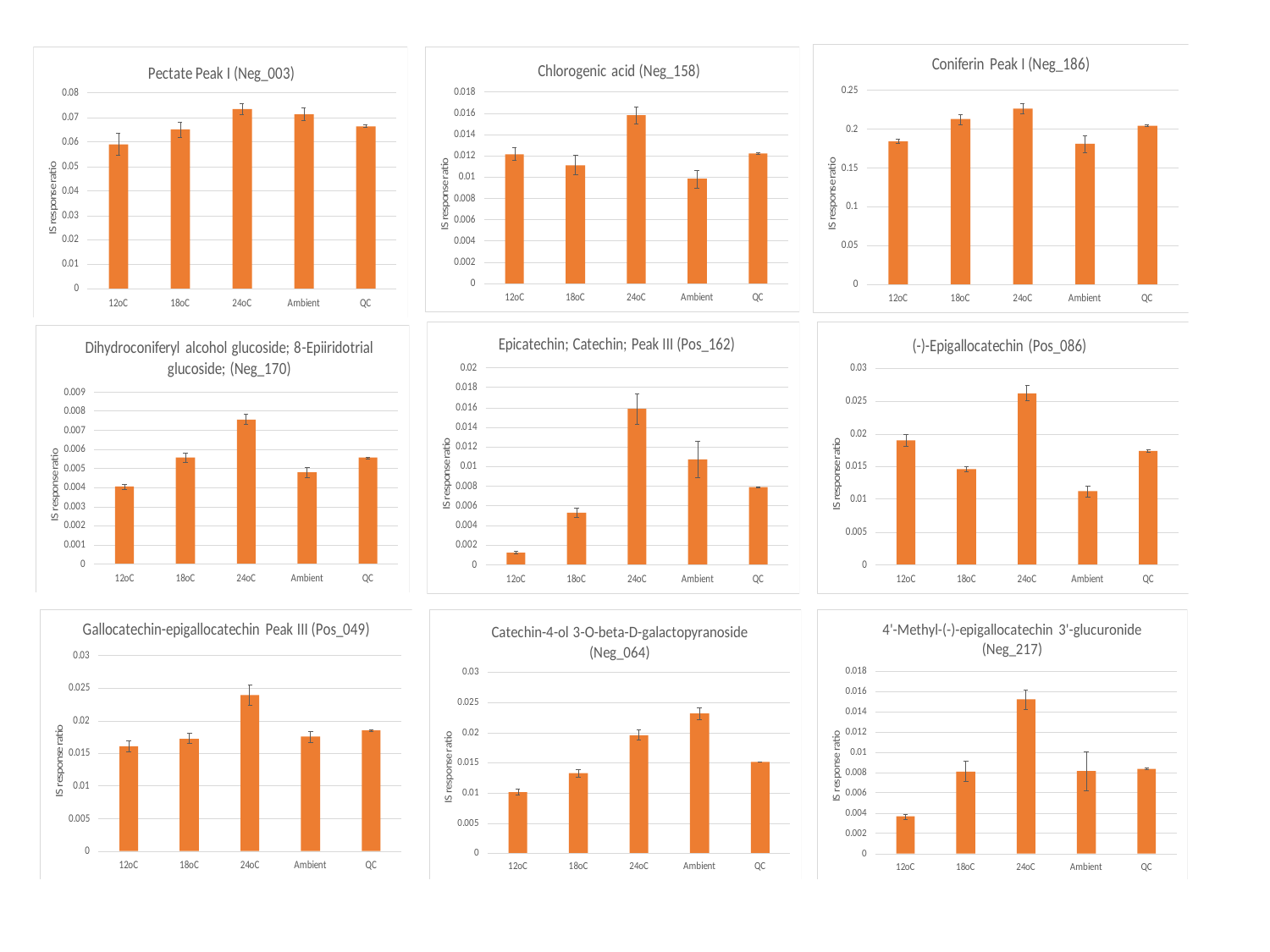

## Slide 3
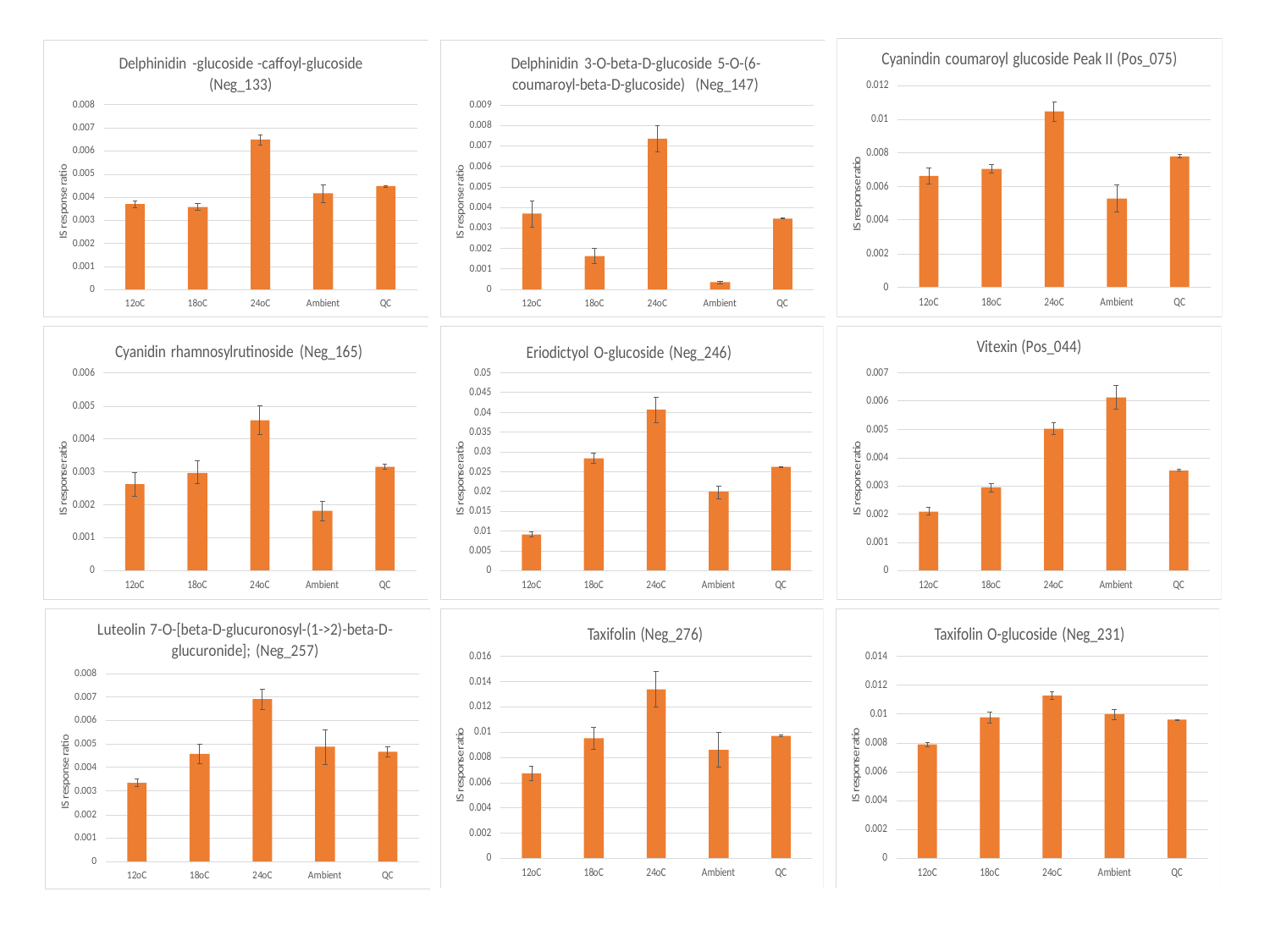

## Slide 4
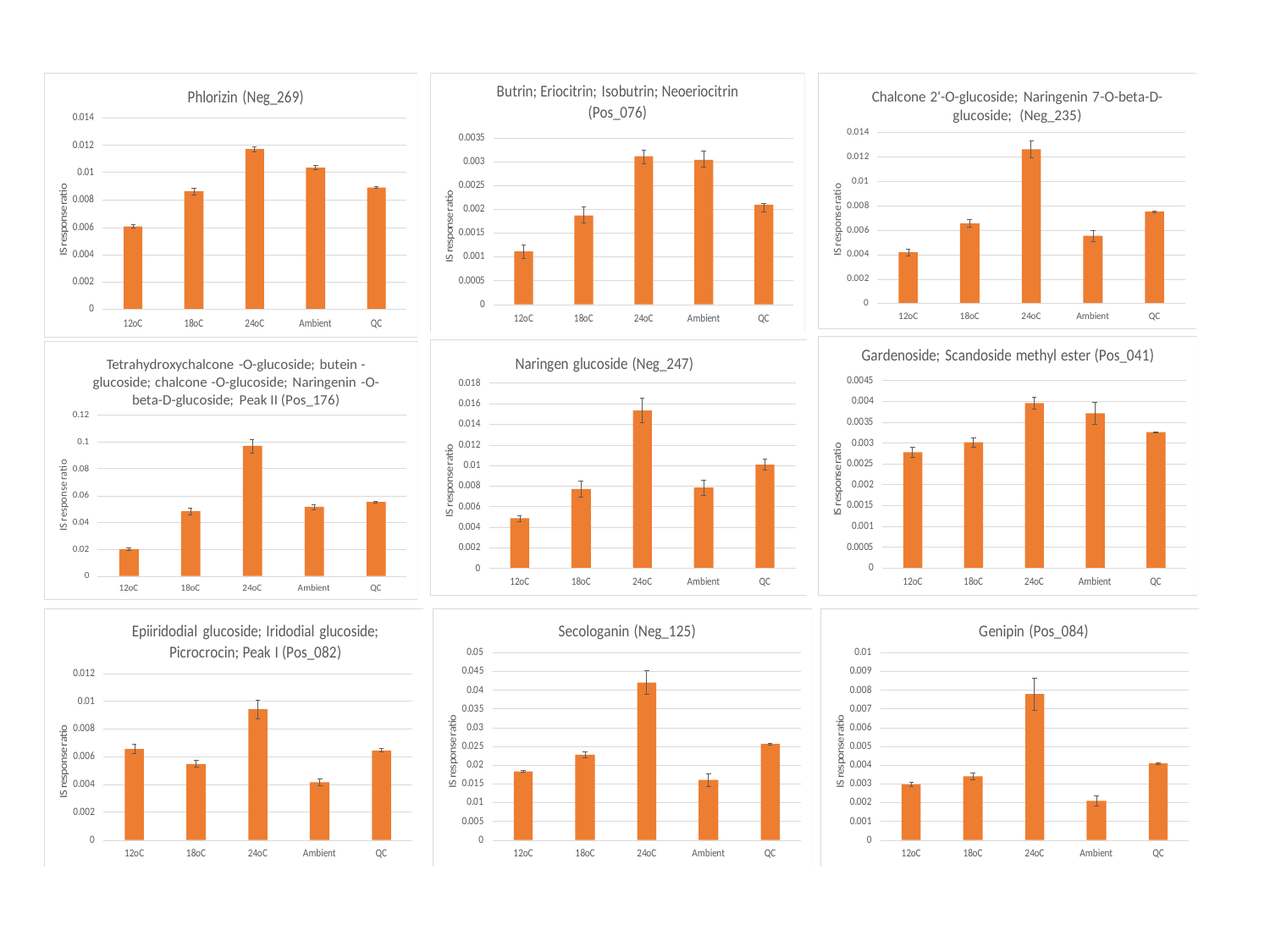

Supplement: Supplementary file 2 — Supplementary material 2 (PPTX 197 KB) Figure S2: Bar charts of all metabolites (not shown in Figure 3) that were elevated under increased growth temperatures. Error bars represent the standard error. [file 11306_2018_1462_MOESM2_ESM.pptx]

## Slide 1
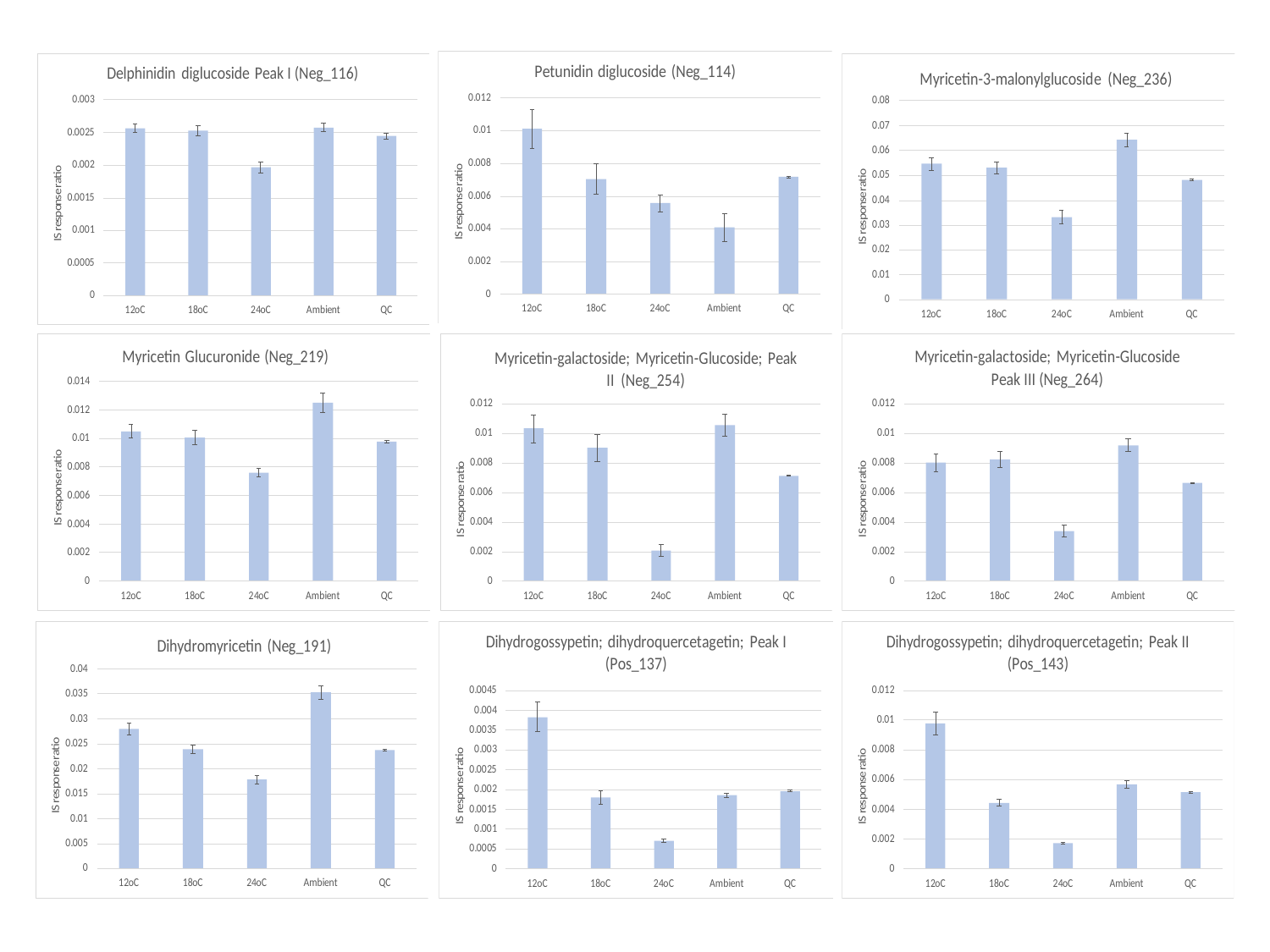

## Slide 2
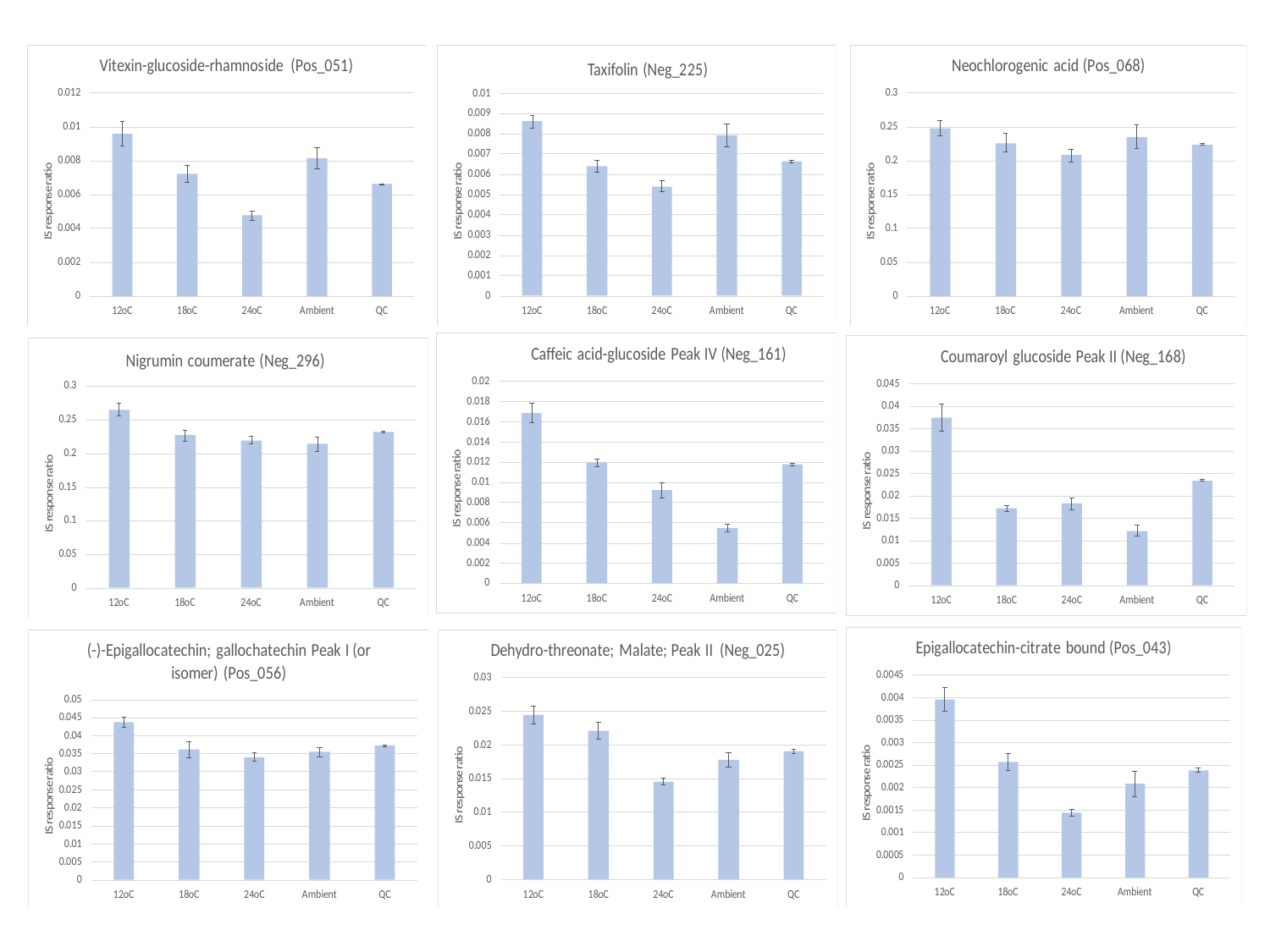

Supplement: Supplementary file 3 — Supplementary material 3 (PPTX 113 KB) Figure S3: Bar charts of all metabolites (not shown in Figure 4) that were reduced under increased growth temperatures. Error bars represent the standard error. [file 11306_2018_1462_MOESM3_ESM.pptx]

## Slide 1
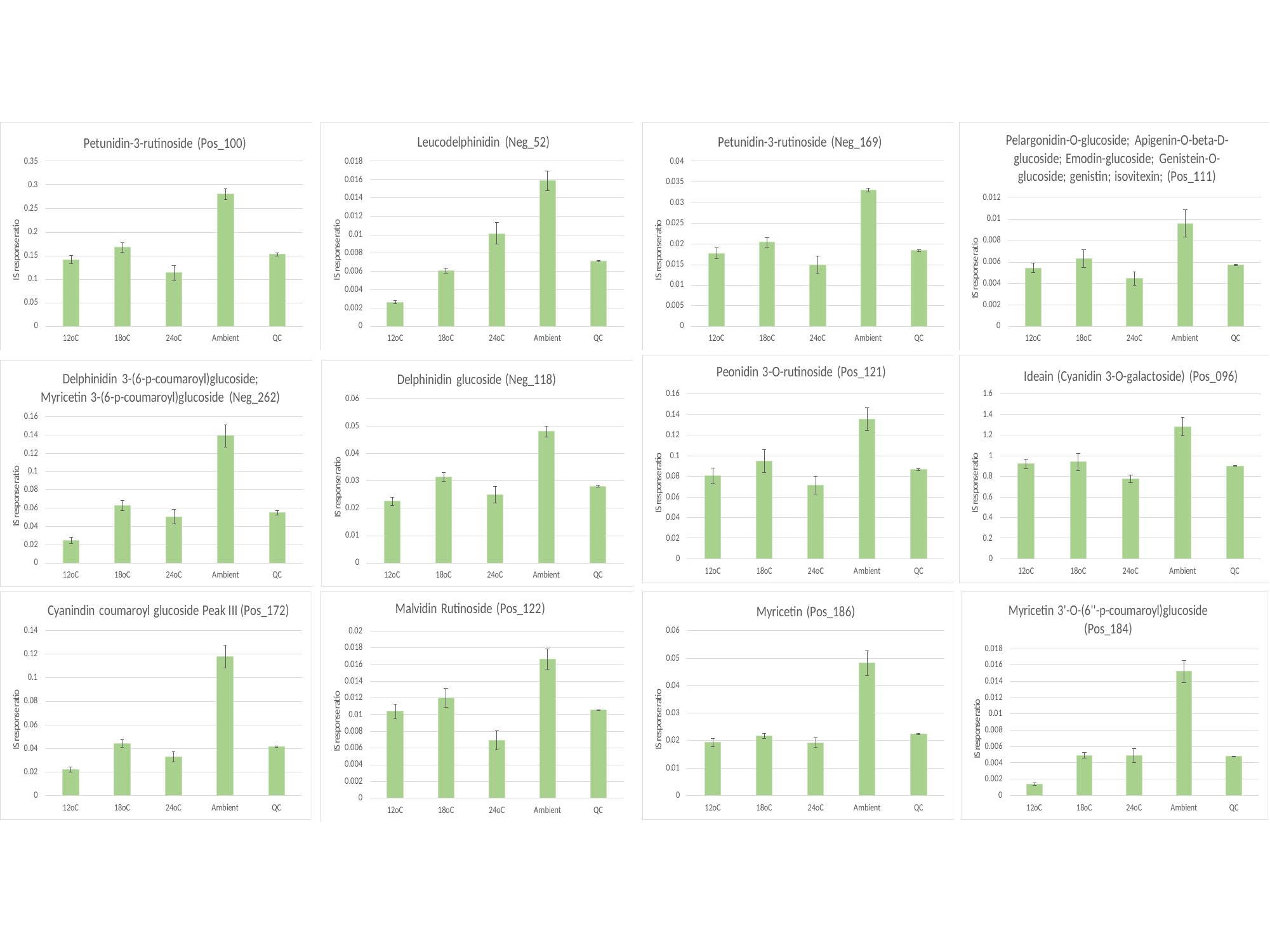

## Slide 2
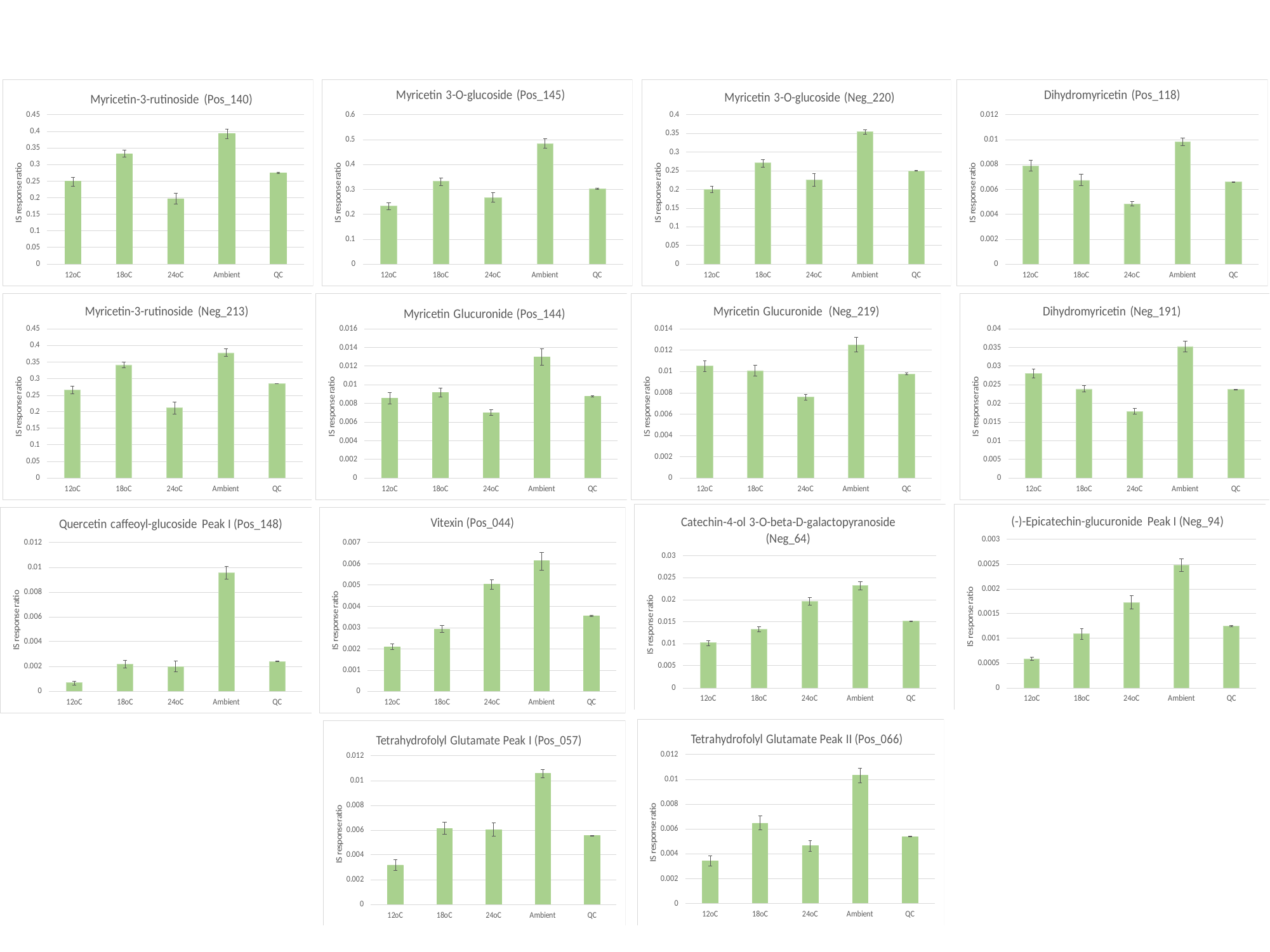

Supplement: Supplementary file 4 — Supplementary material 4 (PPTX 151 KB) Figure S4: Bar charts of all metabolites (not shown in Figure 5a) that were elevated in ambient growth conditions. Error bars represent the standard error. [file 11306_2018_1462_MOESM4_ESM.pptx]

## Slide 1
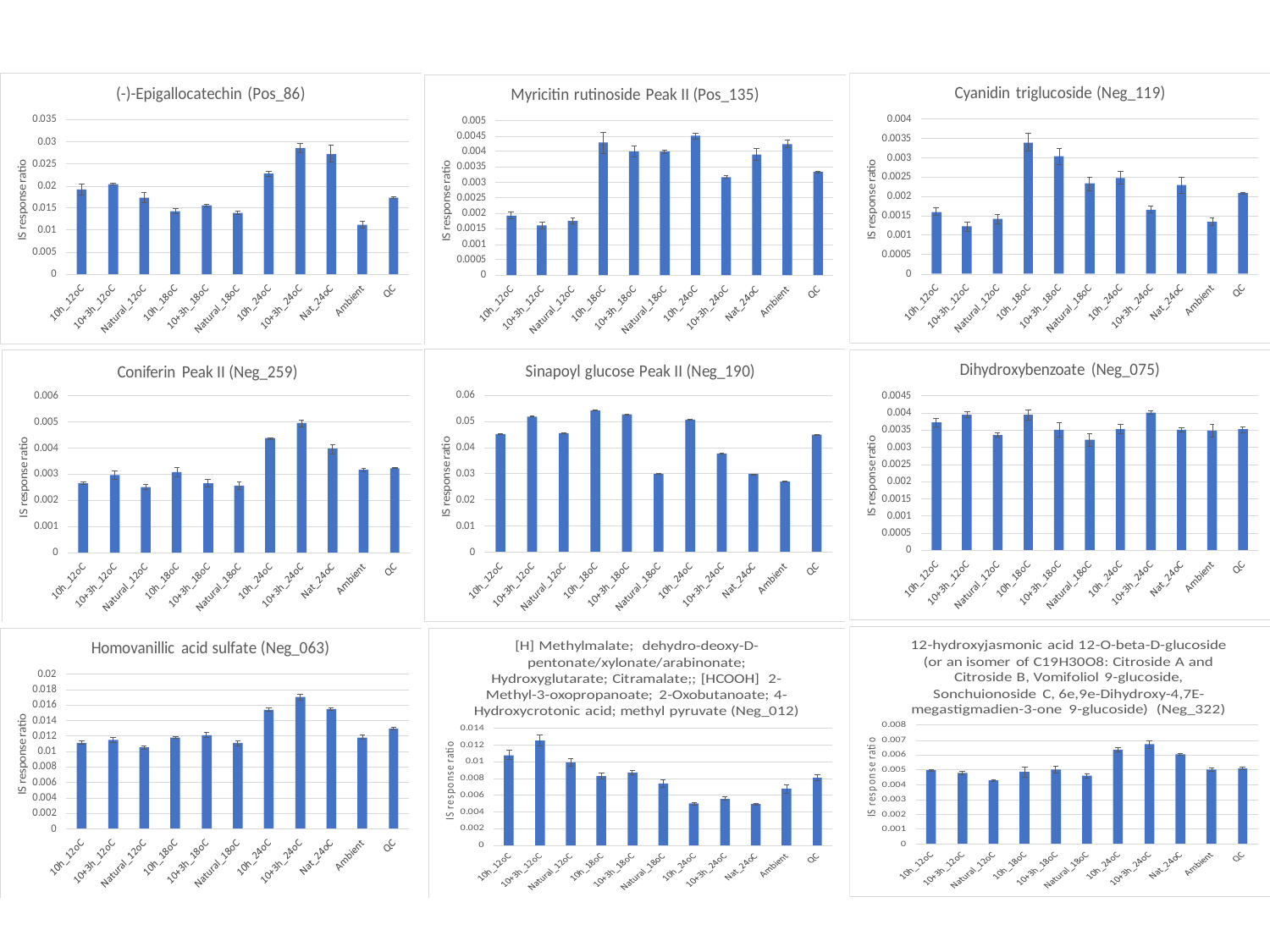

Supplement: Supplementary file 5 — Supplementary material 5 (PPTX 633 KB) Figure S5: Bar charts of all metabolites (not shown in Figure 5b) that were affected by day light conditions. Natural, 10 h, 10 h + 3 h, refer to the following daylength condition descriptions, (1) natural long summer day (LD), ca. 18 h (natural LD), (2) 10 h artificial short day (SD), and (3) 10 h SD + 3 h night interruption (SD + NI), respectively. [file 11306_2018_1462_MOESM5_ESM.pptx]
